# The two-dimensional thio­phosphate CsCrP_2_S_7_

**DOI:** 10.1107/S1600536810030655

**Published:** 2010-08-11

**Authors:** Kyounghee Kim, Jooran Na, Hoseop Yun

**Affiliations:** a Division of Energy Systems Research and Department of Chemistry, Ajou University, Suwon 443-749, Republic of Korea

## Abstract

The quaternary title compound, caesium chromium(III) hepta­thio­diphosphate(V), CsCrP_2_S_7_, has been synthesized using the reactive halide flux method. It is isotypic with other *AM*P_2_S_7_ (*A* = alkali metal; *M* = Cr, V or In) structures and consists of two-dimensional _∞_^2^[CrP_2_S_7_]^−^ layers extending parallel to (001) which are separated from each other by Cs^+^ ions (symmetry 2). The layer is built up from slightly distorted octa­hedral [CrS_6_] units (symmetry 2) and bent [P_2_S_7_] units consisting of two corner-sharing [PS_4_] tetra­hedra. The [CrS_6_] octa­hedra share two edges and two corners with the [PS_4_] tetra­hedra. There are only van der Waals inter­actions present between the layers. The Cs^+^ ions are located in this van der Waals gap and stabilize the structure through weak ionic inter­actions. The classical charge balance of the title compound can be expressed as [Cs^+^][Cr^3+^][P^5+^]_2_[S^2−^]_7._

## Related literature

For *AM*P_2_S_7_-related quaternary thio­phosphates, see: Kopnin *et al.* (2000 ▶) for K*M*P_2_S_7_ (*M* = Cr, V, In); Durand *et al.* (1993 ▶) for RbVP_2_S_7_; Gutzmann *et al.* (2005 ▶) for CsVP_2_S_7_. For the related mixed-metallic phase KV_1-*x*_Cr_*x*_P_2_S_7_, see: Sekizawa *et al.* (2004 ▶). Related structures were reported by Coste *et al.* (2001 ▶); Derstroff *et al.* (2002 ▶); Toffoli *et al.* (1982 ▶); Wang *et al.* (1989 ▶).

## Experimental

### 

#### Crystal data

CrCsP_2_S_7_
*M* *_r_* = 471.34
Monoclinic, 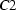

*a* = 8.5867 (7) Å
*b* = 9.5461 (7) Å
*c* = 6.7504 (6) Å
β = 97.572 (3)°
*V* = 548.50 (8) Å^3^
*Z* = 2
Mo *K*α radiation
μ = 5.87 mm^−1^
*T* = 290 K
0.14 × 0.02 × 0.02 mm

#### Data collection

Rigaku R-AXIS RAPID diffractometer
Absorption correction: multi-scan (*ABSCOR*; Higashi, 1995 ▶) *T*_min_ = 0.608, *T*_max_ = 1.000
2714 measured reflections
1268 independent reflections
1052 reflections with *I* > 2σ(*I*)
*R*_int_ = 0.039

#### Refinement

*R*[*F* ^2^ > 2σ(*F* ^2^)] = 0.029
*wR*(*F* ^2^) = 0.073
*S* = 1.18
1268 reflections
52 parameters
1 restraint
Δρ_max_ = 1.01 e Å^−3^
Δρ_min_ = −1.09 e Å^−3^
Absolute structure: Flack (1983 ▶), 593 Friedel pairs
Flack parameter: −0.01 (3)

### 

Data collection: *RAPID-AUTO* (Rigaku, 2006 ▶); cell refinement: *RAPID-AUTO*; data reduction: *RAPID-AUTO*; program(s) used to solve structure: *SHELXS97* (Sheldrick, 2008 ▶); program(s) used to refine structure: *SHELXL97* (Sheldrick, 2008 ▶); molecular graphics: locally modified version of *ORTEP* (Johnson, 1965 ▶); software used to prepare material for publication: *WinGX* (Farrugia, 1999 ▶).

## Supplementary Material

Crystal structure: contains datablocks global, I. DOI: 10.1107/S1600536810030655/wm2382sup1.cif

Structure factors: contains datablocks I. DOI: 10.1107/S1600536810030655/wm2382Isup2.hkl

Additional supplementary materials:  crystallographic information; 3D view; checkCIF report

## Figures and Tables

**Table:** 

Cr—S1	2.414 (2)
Cr—S2	2.440 (2)
Cr—S3^i^	2.430 (2)
P—S1	2.025 (3)
P—S2	2.024 (3)
P—S3	2.012 (2)
P—S4	2.134 (2)

**Table:** 

P^ii^—S4—P	107.50 (14)

Symmetry codes: (i) 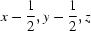; (ii) 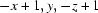.

**Fig. 1.**
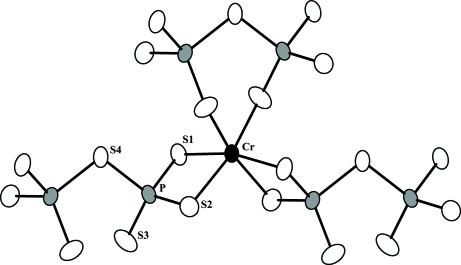
The interconnection mode of the [CrS6] octahedron and the [P2S74-] units with the atom labelling scheme. Displacement ellipsoids are drawn at the 80% probability level.

**Fig. 2.**
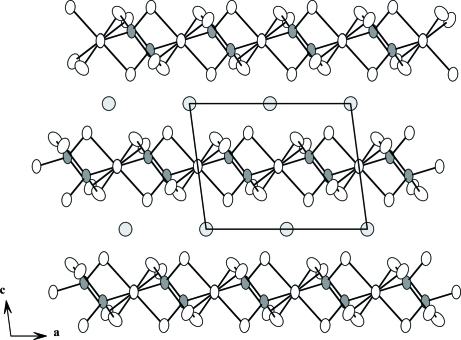
A perspective view of CsCrP2S7 down the b axis showing the stacking of the layers. Filled, dark gray, pale gray, and open circles represent Cr, P, Cs, and S atoms, respectively. The Cs—S bonds are omitted for clarity. The displacement ellipsoids are drawn at the 80% probability level.

## supplementary crystallographic information

### Comment

During an attempt to prepare new chromium thiophosphates with the use of halide fluxes, a new compound was isolated. Here we report the synthesis and structure of the new layered quaternary thiophosphate, CsCrP_2_S_7_.

The title compound is a new member of the previously reported isotypic *AM*P_2_S_7_ family (*A* = alkali metal; *M* = Cr, V, or In) (Kopnin *et al.*, 2000; Durand *et al.*, 1993; Gutzmann *et al.*, 2005, Sekizawa *et al.* , 2004). The structure of CsCrP_2_S_7_ consists of layers with composition _∞_^2^[CrP_2_S_7_]^-^ which are composed of [CrS_6_] octahedra and bent [P_2_S_7_^4-^] units made up of two corner-sharing [PS_4_] tetrahedra. As usually found in other chromium sulfides (Derstroff *et al.,* 2002), each Cr atom is surrounded by six S atoms in a (slightly distorted) octahedral arrangement. In the title compound they share two edges and two corners with the [PS_4_] tetrahedra to form the two-dimensional infinite layer extending parallel to (001) (Fig. 1). There are only van der Waals interactions between the layers and the Cs^+^ ions in this van der Waals gap stabilize the structure through weak ionic interactions (Fig. 2).

While both the [CrS_6_] octahedron and the [PS_4_] tetrahedron show angular distortions, the Cr—S and P—S distances are rather regular and in good agreement with those found in other related phases (e.g. Coste *et al.*, 2001). Atom S4 is bridging two P atoms in the [P_2_S_7_^4-^] units. The bridging P—S4 bond is longer than those of the terminal bonds, a characteristic feature for two condensed PS_4_ tetrahedra (Toffoli *et al.*, 1982) or PO_4_ tetrahedra (Wang *et al.*, 1989).

The Cs^+^ ion is surrounded by twelve S atoms if an arbitrarily choosen cut-off of 4.2 Å for the Cs—S bonding interactions is used. The anharmonic behavior of the alkali metal ion, as observed in the isotypic K or Rb analogues (Kopnin *et al.*, 2000; Durand *et al.*, 1993), is not found here. The harmonic behavior of the Cs^+^ ion in the title compound could be due to the larger ionic radius and hence to a larger coordination number (Gutzmann *et al.*, 2005). The classical charge balance of the title compound can be expressed as [Cs^+^][Cr^3+^][[P^5+^]_2_[S^2-^]_7_.

### Experimental

The title compound, CsCrP_2_S_7_, was prepared by the reaction of the elemental Cr, P and S with the use of the reactive alkali metal halide flux technique. A combination of the pure elements, Cr powder (CERAC 99.95%), P powder (CERAC 99.5%) and S powder (Aldrich 99.999%) were mixed in a fused silica tube in a molar ratio of Cr: P: S = 1: 2: 6 with CsCl/LiCl. The mass ratio of the reactants and the alkali halide flux was 1: 3. The tube was evacuated to 0.133 Pa, sealed and heated gradually (50 K/h) to 923 K, where it was kept for 72 h. The tube was cooled to 473 K at 3 K/h and then was quenched to room temperature. The excess halides were removed with distilled water and dark brown needle shaped crystals were obtained. The crystals are stable in air and water. Semi-qualitative analysis of the crystals with XRF indicated the presence of Cs, Cr, P, and S. No other element was detected.

### Refinement

A difference Fourier synthesis calculated with phase based on the final parameters shows that the highest residual electron density (1.01 e/Å^3^) is 0.89 Å from the Cs site and the deepest hole (-1.09 e/Å^3^) is 1.74 Å from the S3 site.

### Crystal data

**Table:**

CrCsP_2_S_7_	*F*(000) = 442
*M**_r_* = 471.34	*D*_x_ = 2.854 Mg m^−^^3^
Monoclinic, *C*2	Mo *K*α radiation, λ = 0.71073 Å
Hall symbol: C 2y	Cell parameters from 10764 reflections
*a* = 8.5867 (7) Å	θ = 3.0–27.5°
*b* = 9.5461 (7) Å	µ = 5.87 mm^−^^1^
*c* = 6.7504 (6) Å	*T* = 290 K
β = 97.572 (3)°	Needle, dark brown
*V* = 548.50 (8) Å^3^	0.14 × 0.02 × 0.02 mm
*Z* = 2	

### Data collection

**Table:**

Rigaku R-AXIS RAPID diffractometer	1052 reflections with *I* > 2σ(*I*)
graphite	*R*_int_ = 0.039
ω scans	θ_max_ = 27.5°, θ_min_ = 3.0°
Absorption correction: multi-scan (*ABSCOR*; Higashi, 1995)	*h* = −10→11
*T*_min_ = 0.608, *T*_max_ = 1.000	*k* = −12→12
2714 measured reflections	*l* = −8→8
1268 independent reflections	

### Refinement

**Table:**

Refinement on *F*^2^	1 restraint
Least-squares matrix: full	*w* = 1/[σ^2^(*F*_o_^2^) + (0.0208*P*)^2^ + 0.2322*P*] where *P* = (*F*_o_^2^ + 2*F*_c_^2^)/3
*R*[*F*^2^ > 2σ(*F*^2^)] = 0.029	(Δ/σ)_max_ < 0.001
*wR*(*F*^2^) = 0.073	Δρ_max_ = 1.01 e Å^−^^3^
*S* = 1.18	Δρ_min_ = −1.09 e Å^−^^3^
1268 reflections	Absolute structure: Flack (1983), 593 Friedel pairs
52 parameters	Flack parameter: −0.01 (3)

### Special details

**Table:**

Geometry. All e.s.d.'s (except the e.s.d. in the dihedral angle between two l.s. planes) are estimated using the full covariance matrix. The cell e.s.d.'s are taken into account individually in the estimation of e.s.d.'s in distances, angles and torsion angles; correlations between e.s.d.'s in cell parameters are only used when they are defined by crystal symmetry. An approximate (isotropic) treatment of cell e.s.d.'s is used for estimating e.s.d.'s involving l.s. planes.

### Fractional atomic coordinates and isotropic or equivalent isotropic displacement parameters (Å^2^)

**Table:**

	*x*	*y*	*z*	*U*_iso_*/*U*_eq_	
Cs	0.5	0.39716 (9)	0	0.0432 (2)	
Cr	0	0.48837 (14)	0.5	0.0198 (4)	
P	0.3006 (2)	0.63947 (16)	0.4247 (3)	0.0188 (4)	
S1	0.15938 (19)	0.51649 (17)	0.2337 (3)	0.0235 (4)	
S2	0.1839 (2)	0.65997 (17)	0.6648 (3)	0.0272 (5)	
S3	0.3619 (2)	0.81248 (18)	0.2834 (3)	0.0283 (5)	
S4	0.5	0.5073 (3)	0.5	0.0218 (5)	

### Atomic displacement parameters (Å^2^)

**Table:**

	*U*^11^	*U*^22^	*U*^33^	*U*^12^	*U*^13^	*U*^23^
Cs	0.0254 (4)	0.0753 (6)	0.0289 (4)	0	0.0042 (3)	0
Cr	0.0113 (8)	0.0170 (7)	0.0317 (10)	0	0.0048 (7)	0
P	0.0110 (9)	0.0179 (8)	0.0274 (11)	−0.0028 (6)	0.0022 (8)	−0.0004 (8)
S1	0.0149 (8)	0.0271 (9)	0.0291 (10)	−0.0051 (7)	0.0053 (8)	−0.0056 (8)
S2	0.0235 (11)	0.0249 (9)	0.0355 (12)	−0.0039 (7)	0.0127 (9)	−0.0090 (9)
S3	0.0258 (10)	0.0247 (9)	0.0319 (11)	−0.0102 (8)	−0.0056 (9)	0.0070 (9)
S4	0.0119 (11)	0.0249 (13)	0.0288 (15)	0	0.0039 (10)	0

### Geometric parameters (Å, °)

**Table:**

Cr—S1^i^	2.414 (2)	P—S1	2.025 (3)
Cr—S1	2.414 (2)	P—S2	2.024 (3)
Cr—S2	2.440 (2)	P—S3	2.012 (2)
Cr—S2^i^	2.440 (2)	P—S4	2.134 (2)
Cr—S3^ii^	2.430 (2)	S3—Cr^iv^	2.430 (2)
Cr—S3^iii^	2.430 (2)	S4—P^v^	2.134 (2)
			
S1^i^—Cr—S1	167.23 (10)	S3^iii^—Cr—S2^i^	166.77 (6)
S1^i^—Cr—S3^ii^	104.20 (7)	S2—Cr—S2^i^	95.64 (10)
S1—Cr—S3^ii^	84.74 (7)	S3—P—S2	119.23 (11)
S1^i^—Cr—S3^iii^	84.74 (7)	S3—P—S1	110.24 (12)
S1—Cr—S3^iii^	104.20 (7)	S2—P—S1	104.37 (10)
S3^ii^—Cr—S3^iii^	92.60 (9)	S3—P—S4	110.27 (11)
S1^i^—Cr—S2	88.98 (7)	S2—P—S4	109.45 (11)
S1—Cr—S2	82.44 (6)	S1—P—S4	101.73 (10)
S3^ii^—Cr—S2	166.77 (6)	P—S1—Cr	86.60 (9)
S3^iii^—Cr—S2	87.38 (7)	P—S2—Cr	85.93 (9)
S1^i^—Cr—S2^i^	82.44 (6)	P—S3—Cr^iv^	114.79 (11)
S1—Cr—S2^i^	88.98 (7)	P^v^—S4—P	107.50 (14)
S3^ii^—Cr—S2^i^	87.38 (7)		

Symmetry codes: (i) −*x*, *y*, −*z*+1; (ii) *x*−1/2, *y*−1/2, *z*; (iii) −*x*+1/2, *y*−1/2, −*z*+1; (iv) *x*+1/2, *y*+1/2, *z*; (v) −*x*+1, *y*, −*z*+1.
